# Nanoscale Organization, Regulation, and Dynamic Reorganization of Cardiac Calcium Channels

**DOI:** 10.3389/fphys.2021.810408

**Published:** 2022-01-05

**Authors:** Rose E. Dixon

**Affiliations:** Department of Physiology and Membrane Biology, School of Medicine, University of California, Davis, Davis, CA, United States

**Keywords:** CaV1.2 Ca^2+^ channel, RyR2, EC-coupling, heart failure, β-adrenergic regulation, L-type calcium channels, ion channel clustering

## Abstract

The architectural specializations and targeted delivery pathways of cardiomyocytes ensure that L-type Ca^2+^ channels (CaV1.2) are concentrated on the t-tubule sarcolemma within nanometers of their intracellular partners the type 2 ryanodine receptors (RyR2) which cluster on the junctional sarcoplasmic reticulum (jSR). The organization and distribution of these two groups of cardiac calcium channel clusters critically underlies the uniform contraction of the myocardium. Ca^2+^ signaling between these two sets of adjacent clusters produces Ca^2+^ sparks that in health, cannot escalate into Ca^2+^ waves because there is sufficient separation of adjacent clusters so that the release of Ca^2+^ from one RyR2 cluster or supercluster, cannot activate and sustain the release of Ca^2+^ from neighboring clusters. Instead, thousands of these Ca^2+^ release units (CRUs) generate near simultaneous Ca^2+^ sparks across every cardiomyocyte during the action potential when calcium induced calcium release from RyR2 is stimulated by depolarization induced Ca^2+^ influx through voltage dependent CaV1.2 channel clusters. These sparks summate to generate a global Ca^2+^ transient that activates the myofilaments and thus the electrical signal of the action potential is transduced into a functional output, myocardial contraction. To generate more, or less contractile force to match the hemodynamic and metabolic demands of the body, the heart responds to β-adrenergic signaling by altering activity of calcium channels to tune excitation-contraction coupling accordingly. Recent accumulating evidence suggests that this tuning process also involves altered expression, and dynamic reorganization of CaV1.2 and RyR2 channels on their respective membranes to control the amplitude of Ca^2+^ entry, SR Ca^2+^ release and myocardial function. In heart failure and aging, altered distribution and reorganization of these key Ca^2+^ signaling proteins occurs alongside architectural remodeling and is thought to contribute to impaired contractile function. In the present review we discuss these latest developments, their implications, and future questions to be addressed.

## Introduction

**Figure 1:**
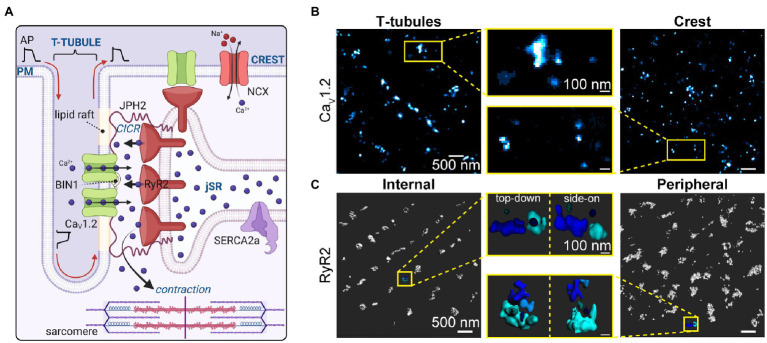
Calcium channel organization/clustering in the Dyad. **(A)** Illustration depicting the cardiac dyad where CaV1.2 channels are delivered along microtubules to BIN1-hubs on the t-tubule membrane. There they concentrate and form clusters of variable sizes, opposite clusters of RyR2 on the junctional sarcoplasmic reticulum. The dyad is held together by JPH2 which anchors the jSR within 12–15 nm of the t-tubule sarcolemma. JPH2 interacts with both CaV1.2 and RyR2, affecting RyR2 gating, and potentially playing a role in CaV1.2 retention in the dyad. Not all CaV1.2 channels and RyR2 are found in these t-tubule:jSR junctional complexes, some are found in junctions at the sarcolemmal crest and others (not illustrated) are found at junctions along longitudinally oriented axial-tubules. **(B)** CaV1.2 channel clusters at the t-tubule (*left*) and crest (*right*) sarcolemma are seen in super-resolution Ground State Depletion (GSD) localization maps from fixed, immunostained mouse ventricular myocytes. Yellow boxes indicate zoomed-in regions (*middle*). Reproduced and modified with permission from (Del Villar et al., 2021). **(C)** RyR2 clusters are observed in 3D-renderings of 3D-STORM images of internal and peripherally located RyR2s in fixed, immunostained rat cardiomyocytes. Yellow boxes indicate zoomed-in regions of each image from top-down, and side-on perspectives. Panel C was reproduced and modified with permission from (Shen et al., 2019). Illustrations were created in *Biorender*.

During action potential (AP)-driven membrane depolarization in ventricular myocytes, Ca^2+^ influx across the plasma membrane occurs through voltage-gated L-type CaV1.2 channels. The level of calcium influx through CaV1.2 channels triggers a graded amount of calcium induced calcium release (CICR) from the type 2 ryanodine receptors (RyR2) on the juxtaposed junctional sarcoplasmic reticulum (jSR) at specialized nanodomains between the sarcoplasmic reticulum (SR) and the t-tubule or surface plasma membrane (PM). These SR-PM junctions termed dyads or calcium release units (CRUs), house the fundamental ion channel machinery for EC-coupling, bringing clusters of CaV1.2 channels into nanometer proximity of their intracellular Ca^2+^ channel partners RyR2 (Figure 1). These two cardiac calcium channel partners participate in feedforward and feedback signaling to permit the faithful beat-to-beat coupling of electrical excitation to Ca^2+^-driven mechanical contraction. Here we review the nanoscale organization of CaV1.2 and RyR2 channels, and how it can be regulated by a key stress signaling pathway (β-adrenergic receptor (β-AR) signaling). We also highlight nanoscale reorganization of these two calcium channels that occurs during heart failure (HF) and aging and discuss the implications for contractile function.

## CaV1.2 Channel Localization in Ventricular Myocytes

CaV1.2 are heteromeric channel complexes consisting of a pore-forming and voltage-sensing α_1C_ subunit (~250 kDa), associated with CaVβ (~52–72 kDa depending on the isoform), CaVα_2_δ (~175 kDa), and sometimes CaVγ (~32 kDa) auxiliary subunits in a 1:1:1:1 stoichiometry (Catterall, 2011; Zamponi et al., 2015; Dolphin, 2016; Westhoff and Dixon, 2021). To directly influence EC-coupling, CaV1.2 channels must be located at dyadic junctions in the vicinity of the sarcomeres where the influx of Ca^2+^ can be coupled to CICR from RyR2 on the jSR. Dyads are predominantly found at interfaces between the jSR and the t-tubule membrane, but there are a significant number of functional dyads on the axial/longitudinal tubule interfaces and at the sarcolemmal crest interface (Asghari et al., 2009). It should be noted that despite the continued use of t-tubules as the standard nomenclature for the non-surface, tubular network of sarcolemma, a convention we maintain herein, around 40% of the network is not transverse at all but rather lies longitudinally or axially along the length of the cell between the z-lines in rat ventricular myocytes (Soeller and Cannell, 1999), although the proportion of longitudinally-orientated tubules varies between species (Setterberg et al., 2021).

In health, the vast majority of CaV1.2 channels are located on the t-tubule membranes (Scriven et al., 2000) where the channel density is around 9 times that of the surface sarcolemma (Bers, 2001). This preferential t-tubular distribution of CaV1.2 channels is apparent in immuno-stained myocytes using conventional fluorescence (Carl et al., 1995) and super-resolution imaging approaches (Dixon et al., 2015; Ito et al., 2019; Del Villar et al., 2021). Detubulation of rat ventricular myocytes with formamide, an approach that severs the transverse and axial tubules but allows their openings at the surface sarcolemma to reseal, leads to loss of ~25% of the cell capacitance but an ~75% reduction in *I*_Ca_, indicating that the t-tubular membrane constitutes around a quarter of the total sarcolemmal area but houses almost three quarters of the functional CaV1.2 channels (Kawai et al., 1999). The remaining 25% of CaV1.2 represent the surface membrane subpopulation of channels which are thought to play a critical role in SR Ca^2+^ loading since detubulination does not significantly affect SR Ca^2+^ load.

Super-resolution single molecule localization microscopy (SMLM) has revealed that CaV1.2 channels are not just homogenously distributed along the sarcolemma but rather, preferentially form clusters in both the t-tubules (Dixon et al., 2015, 2017; Ito et al., 2019; Del Villar et al., 2021) and sarcolemmal crest of ventricular myocytes (Del Villar et al., 2021; see Figure 1B), as well as human embryonic stem cell derived cardiomyocytes (hESC-CMs; De La Mata et al., 2019). Stepwise photobleaching experiments have been used to quantify the number of channels per cluster, likely limited to the surface sarcolemma since these experiments were performed in TIRF (Dixon et al., 2015; Ito et al., 2019). In those studies, myocytes were transduced with photoactivatable GFP-tagged CaVβ subunits which bind to CaVα_1C_ with a 1:1 stoichiometry allowing them to function as fluorescent biosensors of channel location when they are photoactivated. Using this approach, the mean number of channels per surface cluster is 6–8 although this is likely a gross underestimate given that most channels are likely bound to endogenous, non-fluorescent CaVβ and remain uncounted. Cryo-EM structures of the related skeletal muscle L-type Ca^2+^ channel, CaV1.1 have revealed the widest aspect of the channel as 100 Å (10 nm; Wu et al., 2015, 2016). Assuming a single CaV1.2 channel occupies a similar maximum area of 100 nm^2^, then t-tubule clusters may contain on the order of ~20–37 channels based on published cluster areas. Crest cluster areas have only been measured in one study thus far but were not significantly different from t-tubule cluster areas (Del Villar et al., 2021) so a similar channel content may be inferred there. In both instances, these estimates assume tight packing of channels with no space between them and are thus likely overestimates. Future studies are needed to accurately quantify the number of channels per cluster. As discussed in Section “RyR2 Localization in Ventricular Myocytes”, cluster size can be regulated by signal transduction pathways including β-adrenergic signaling, with repercussions for channel function, Ca^2+^ influx, and inotropy (Ito et al., 2019; Del Villar et al., 2021).

Targeted delivery of CaV1.2 to the t-tubule membrane is thought to occur *via* microtubules anchored by a protein called bridging integrator 1 (BIN1; Hong et al., 2010, 2012). BIN1 also plays a role in the formation of t-tubules which relies on its ability to curve membranes upon dimerization of its N-terminal BAR domain (Lee et al., 2002; Frost et al., 2009; Hong et al., 2010). Channel conveyance to BIN1 delivery hubs on the membrane is, however not dependent on membrane tubulation as demonstrated by the abrogation of CaV1.2 delivery in cells expressing a truncated BIN1(1–282) which retains the BAR domain and tubulation ability but lacks the coiled-coil and SH3 domains that form the remainder of the full-length BIN1 structure (Hong et al., 2010). This implies that targeted delivery of CaV1.2 is reliant on the coiled-coil and/or SH3 domains of BIN1. In addition, a cardiac specific isoform of BIN1 (BIN1 + 13 + 17) is thought to generate microfolds on the t-tubule membrane which support the formation of diffusion-restricted microdomains and facilitate clustering of CaV1.2 channels already at the t-tubules (Hong et al., 2014). The role of BIN1 in CaV1.2 and RyR2 displacement and mis-regulation during cardiac pathology is discussed in detail in section “Role of Bridging Integrator 1” below.

Non-dyadic CaV1.2 channel subpopulations are present in caveolae, and non-caveolar lipid rafts as reviewed elsewhere (Maguy et al., 2006; Best and Kamp, 2012). These extra-dyadic populations (Levin and Page, 1980) have been reported to contribute to EC-coupling by affecting the efficiency of Ca^2+^ release from the SR (Calaghan and White, 2006), while other reports suggest they do not contribute directly to EC-coupling (Balijepalli et al., 2006; Correll et al., 2017). Lipid rafts have been proposed to form platforms for dyads in cultured rat ventricular myocytes with the dyadic spacer and SR/PM tethering protein junctophilin-2 (JPH2) associating with cholesterol and the muscle isoform of caveolin (Cav3) to form junctional membrane complexes to which CaV1.2 channels are recruited (Poulet et al., 2021). JPH2 is discussed in greater detail in Section “Role of Junctophilin 2”.

## RyR2 Localization in Ventricular Myocytes

RyR2 are very large (~2.2 MDa) intracellular, Ca^2+^ activated calcium channels found on the ER/SR membrane of most cells (Lanner et al., 2010). These largest known ion channels are homotetrameric assemblies with individual subunits of ~565 kDa (Van Petegem, 2015). Electron microscopy studies have revealed that RyR2 are predominantly, but not exclusively localized to the jSR at t-tubule adjacent locations in junctional complexes/dyads (Franzini-Armstrong et al., 1999). The 3D geometry of RyR2 clusters is variable, complex, and dynamic with them often observed wrapping around t-tubules (Soeller et al., 2007; Hou et al., 2015) and forming larger or smaller clusters depending on their phosphorylation state and/or association with immunophilins FKBP12 or FKBP12.6 (Asghari et al., 2020).

In recent years, super-resolution microscopy techniques have been employed to image RyR2 with 20–30 nm lateral resolution, revealing that the large RyR2 clusters visualized in diffraction-limited confocal microscopy (Chen-Izu et al., 2006) are often ‘superclusters’ composed of several smaller clusters which are proposed to work together as a single CRU (Baddeley et al., 2009; Hou et al., 2015). Delineation of CRU boundaries has varied between publications and investigators with a 3D-STORM study dilating clusters by 50 nm in x, y, and z and then fusing the overlapping clusters to decide on the CRU boundary (Shen et al., 2019) while other 2D studies have assigned clusters to the same CRU if they lie within an edge-to-edge distance of 100 nm (Baddeley et al., 2009; Hou et al., 2015) or 150 nm of each other (MacQuaide et al., 2015). These numbers have stemmed from the *in silico* finding that RyRs located within <100 nm of an activated cluster are exposed to sufficiently high levels of Ca^2+^ (>10 μM) to permit their activation (Sobie et al., 2006). Variation in the reported number of RyRs per CRU from study to study may be partially explained by this inconsistency in CRU boundary delineation (Kolstad et al., 2018).

Clusters present on the coverslip adherent surface of myocytes form peripheral couplons and appear in a double-row pattern visible either side of the z-disk (Chen-Izu et al., 2006), with dSTORM imaging revealing a mean of ~14 RyR2 per cluster (Baddeley et al., 2009), and DNA-PAINT approaches reporting 7–9 RyR2 per peripheral cluster (Jayasinghe et al., 2018; Sheard et al., 2019). Deeper inside the cell, internal clusters are larger and contain ~63 RyR2 on average with >80% of clusters containing ≥100 channels (Hou et al., 2015). More recent 3D super-resolution imaging has suggested that peripheral CRUs contain an average of ~18 and internal CRUs ~23 RyR2 (Shen et al., 2019), with the average cluster containing ~10 channels in the periphery and ~13 channel in the internal fraction (see Figure 1C). While an expansion microscopy study in which expanded samples were imaged on an Airyscan confocal microscope reported peripheral clusters contained ~9 RyR2 on average while internal clusters contained ~8 (Sheard et al., 2019). At both locations, there is a high degree of colocalization between RyR2 and JPH2 confirming their dyadic locale (Baddeley et al., 2009; Jayasinghe et al., 2012; Hou et al., 2015). Size of RyR2 clusters has recently been reported to directly influence Ca^2+^ spark frequency (Galice et al., 2018) and amplitude (Xie et al., 2019). This idea will be revisited later in Section “Nanoscale Re-Organization of RyR2”.

## β-Adrenergic Receptor Signaling Stimulates Alterations in the Function and Nanoscale Distribution of CaV1.2

During episodes of acute stress and exercise, catecholamine binding to β*-*ARs on cardiomyocyte sarcolemmas initiates a cellular signaling cascade resulting in activation of adenylyl cyclase, enhanced cAMP production and a subsequent boost in protein kinase A (PKA) activity and downstream phosphorylation of multiple molecular targets and effectors as recently reviewed (Man et al., 2020). One of those effectors is the cardiac CaV1.2 channel complex. When phosphorylated, CaV1.2 channel open probability (*P*_o_) and gating properties are altered in favor of increased activity and longer mode 2 openings (Tsien et al., 1972; Yue et al., 1990), that has recently been proposed to be due to removal of the inhibitory effects of Rad on CaV1.2 (Liu et al., 2020; Papa et al., 2021).

**Figure 2:**
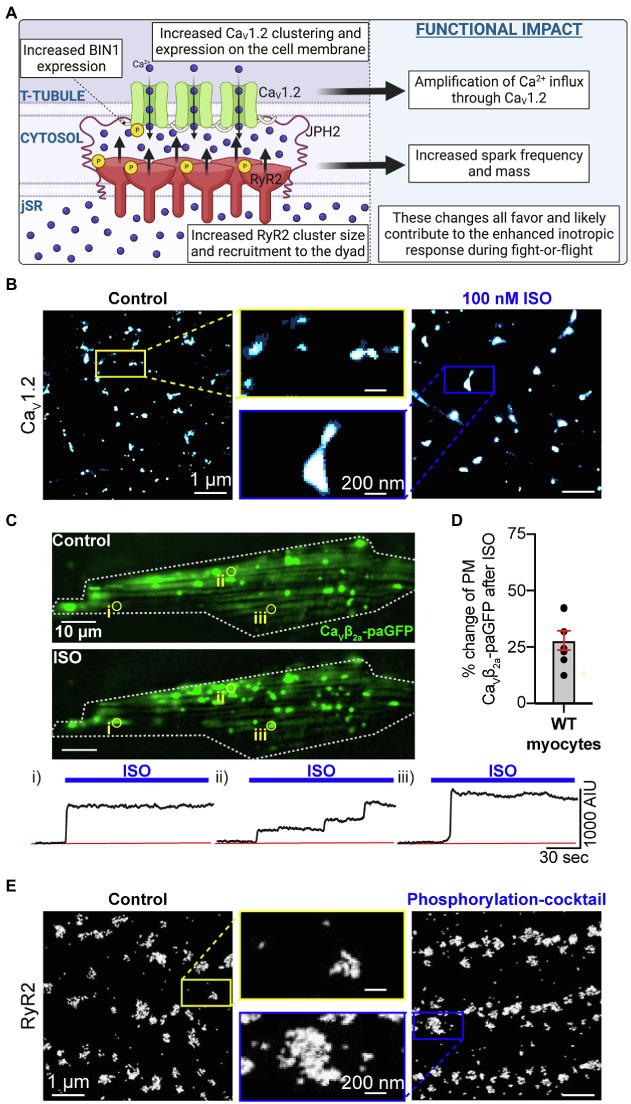
Phosphorylation-mediated expansion of CaV1.2 and RyR2. **(A)** Illustration of phosphorylation-dependent expansion of CaV1.2 and RyR2 in the dyad and its physiological consequences. **(B)** Super-resolution Ground State Depletion (GSD) localization maps from isolated cardiomyocytes immunostained to visualize CaV1.2 channel clusters at the t-tubules in untreated control cells (left) and cells treated with 100 nM ISO for 8 min at room temperature prior to fixation (right). CaV1.2 cluster expansion is evident. **(C**) Show the results of dynamic TIRF imaging performed on mouse ventricular myocytes transduced with AAV9-CaVβ_2_-paGFP which acts as a fluorescent CaV1.2 channel biosensor. The images show the GFP-fluorescence emission from channel located on the surface of a myocyte before and after addition of 100 nM ISO. Individual ROIs are indicated (i, ii, and iii) and show time courses of the changes in GFP-emission at each site where channel clusters are seen to undergo stimulated insertion in response to ISO. **(D**) Histogram summarizing the percentage increase in PM CaVβ_2_-paGFP stimulated by ISO treatment. Panels **B**, **C**, and **D** are reproduced, with permission from (Del Villar et al., 2021). **(E)** Surface RyR2 cluster expansion in response to a phosphorylation cocktail is observed in panel **E** which was reproduced and modified with permission from (Asghari et al., 2020) under the terms of the Creative Commons Attribution 4.0 International Public License (CC-BY 4.0; https://creativecommons.org/licenses/by/4.0/) associated with that article. Illustrations were created in *Biorender*.

Along with the increased channel activity, another aspect of β-adrenergic regulation of CaV1.2 is the apparent increase in the number of functional channels in the membrane. This was first observed in some of the earliest reports of adrenergic regulation of these channels (Sperelakis and Schneider, 1976; Reuter and Scholz, 1977; Bean et al., 1984) but remained unexplained until our recent studies examining CaV1.2 channel dynamics and clustering (Ito et al., 2019; Del Villar et al., 2021). We reported PKA-dependent (H-89 and PKI inhibited), enhanced clustering and expression of CaV1.2 channels on the t-tubule membrane of adult mouse cardiomyocytes after stimulation with ISO (Figures 2A,B). Within these enlarged clusters, channels are packed tightly together, facilitating physical communication between adjacent CaV1.2 such that the opening of one channel in the cluster can drive the opening of other physically interacting channels *via* an allosteric mechanism. This cooperative gating behavior manifests as the simultaneous opening and/or closing of multiple channels. The exact details of the allosteric mechanism are still incompletely understood but is thought to involve Ca^2+^/calmodulin (Ca^2+^•CaM) bridging of neighboring channels *via* their C-terminal tails (Dixon et al., 2015). The overall impact of these interactions between groups of channels in a cluster is an amplification of Ca^2+^ influx into the cell as the highest *P*_o_ channel in the cluster, drives the opening of the others. Since PKA-mediated phosphorylation of CaV1.2 increases channel *P*_o_ an implication of these findings is that a small number of phospho-channels could have a disproportionately large effect as they can drive activity of attached non-phosphorylated channels in their cluster. This effectively increases the number of functional channels, allowing enhanced Ca^2+^ influx during the action potential plateau thus facilitating an inotropic response. Indeed EC-coupling studies of rat ventricular myocytes biolistically transfected with CaV1.2 channels tagged with a light activated dimerization system confirmed that CaV1.2-CaV1.2 interactions and the ensuing amplification of Ca^2+^ influx leads to larger amplitude Ca^2+^ transients indicating enhanced CICR and favoring inotropy (Dixon et al., 2012).

Along with this ISO-stimulated cooperative gating effect on the functional availability of channels, β-AR activation also incites an ~20% increase in the total number of channels in the t-tubule membrane as indicated by super-resolution SMLM studies of CaV1.2, gating current measurements, and biochemical studies of CaV1.2 protein expression (Ito et al., 2019; Del Villar et al., 2021). This ISO-stimulated dynamic augmentation of channel expression was demonstrated in live-cell TIRF experiments on adult mouse cardiomyocytes using a ‘biosensor’ approach whereby a photoactivatable FP-fused CaVβ subunit isoform was packaged into an adeno-associated virus serotype 9 (AAV9) and transduced *in vivo* upon retro-orbital injection of AAV9-CaVβ_2a_-paGFP into anesthetized adult mice (see Figures 2C,D). This smaller (1.8 kb) auxiliary subunit of the channel binds to α_1C_ with a 1:1 stoichiometry; thus, when fused to an FP it can be used as a fluorescent reporter of channel location, albeit a subset of channels since most channels are expected to associate with endogenously expressed CaVβ subunit. Stepwise-photobleaching experiments performed on these transduced myocytes also revealed the presence of larger channel clusters after ISO (Ito et al., 2019). The striking visual of stimulated insertion of CaV1.2 channels into the TIRF footprint of cardiomyocytes invited the question, where are these additional channels coming from?

A subsequent study revealed the presence of a sub-sarcolemmal reservoir of preformed, endosome-localized channels that can be mobilized to the membrane by β-AR stimulation (Del Villar et al., 2021). Mobile vesicular/endosomal CaV1.2 channels had been previously observed in tsA cells (Ghosh et al., 2018), and HL-1 cells (Conrad et al., 2018) but this was the first report of an endosomal reservoir of CaV1.2 channels in primary cardiomyocytes. This internal reserve of channels was posited to contribute to the “functional reserve” of the myocardium that can be accessed during sympathetic activation. Subpopulations of Rab4a-positive early endosomes and Rab11a-positive recycling endosomes were seen to co-localize with CaV1.2 channels, and upon ISO-stimulation the co-localization was reduced coincident with enhanced t-tubule expression of the channels suggesting that the endosomal channels were mobilized to the membrane. SMLM studies revealed the t-tubule membrane as the primary insertion location perhaps related to tighter coupling of the channels with juxta-positioned β*-*AR*s* there (Balijepalli et al., 2006; Chase et al., 2010). Mobilization of these endosomal channels to the membrane was shown to occur along fast Rab4a-dependent and slower Rab11a-dependent recycling pathways which require microtubule polymerization to move the endosomes and their cargo. Given that BIN1, AKA amphiphysin II, anchors microtubules at the t-tubule membrane and is important for t-tubule targeting of CaV1.2, BIN1 expression levels and/or localization may be critical for this stimulated insertion response (Hong et al., 2010). This important hypothesis is currently an open question that should be tested in the future.

## β-Adrenergic Receptor Signaling Stimulates Alterations in the Nanoscale Distribution of RyR2

β-AR stimulation is also associated with reorganization of RyR2 in a manner that appears to involve BIN1-mediated recruitment of phosphorylated-RyR2 (Figure 2A). As discussed above, BIN1 is best known for its role in t-tubule formation and targeted delivery of CaV1.2 channels but there is accumulating evidence that suggests interactions also occur between BIN1 and RyR2. In three-color super-resolution STORM imaging experiments, CaV1.2 and RyR2 clusters were observed with BIN1 ‘sandwiched’ between them (Fu et al., 2016). Along with these immunostaining-observed interactions, CaV1.2 and BIN1 co-immunoprecipitate in HeLa cells (Hong et al., 2010), as do BIN1 and RyR2 in human heart lysates (Fu et al., 2016). β*-*AR stimulation with ISO has been found to produce a rapid augmentation of BIN1 expression in the t-tubules and this is accompanied by an increase in recruitment of phosphorylated RyR2 (p2808-RyR2) to the dyad, shown with diffraction-limited confocal microscopy (Fu et al., 2016). In cardiac specific BIN1 heterozygous (HT) knockout mice, there is of course less BIN1 and so β*-*AR stimulation with ISO increases BIN1 in the t-tubules to a lesser extent than in WT mice, accompanied by a reduced ability to recruit p2808-RyR2 to the dyads. In parallel, ISO-stimulated BIN1^+/−^ HT myocytes were more predisposed to spontaneous Ca^2+^ release events supporting the postulate that the unrecruited pool of p2808-RyR2 may have been left stranded, or ‘orphaned’ in non-dyadic locations, promoting arrhythmogenesis.

In HF, BIN1 expression is reduced, and in the Hong et al. study this was seen to be accompanied by a reduced co-immunoprecipitation of BIN1 with pS2808-RyR2. It is noteworthy however that there is ongoing controversy (addressed in editorial commentaries (Bers, 2012; Valdivia, 2012)) around the original idea from Andy Mark’s lab that pS2808-RyR2 are hyperactive/leaky and enhanced in HF (Marx et al., 2000). Regardless, Hong’s finding of a BIN1-choreographed reorganization of RyR2 upon β-AR stimulation remains an interesting and novel finding and others have since reported phosphorylation dependent increases in RyR2 cluster size in the surface membrane (Asghari et al., 2020; Figure 2E). Future studies should examine whether similar aggregation of RyR2 occurs after phosphorylation in internally localized clusters. In addition, further investigations are required to determine whether BIN1 organizes a similar β*-*AR stimulated recruitment of CaMKII-phosphorylated pS2814-RyR2 to the dyads in healthy and HF models.

## Dyadic Calcium Channel Displacement and Mis-Regulation During Cardiac Pathology

**Figure 3:**
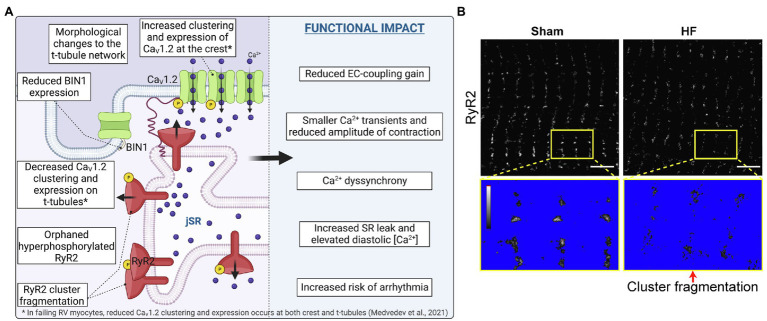
CaV1.2 and RyR2 reorganization and cluster fragmentation in Heart Failure. **(A)** Illustration of the current state of knowledge on CaV1.2 and RyR2 channel reorganization in heart failure. **(B)** dSTORM images of sham-operated control and post-infarction HF rat cardiomyocytes show RyR2 channel cluster fragmentation/dispersal. Yellow boxes indicate zoomed-in regions which are thresholded below to identify channel clusters. Panel B is reproduced and modified with permission from (Kolstad et al., 2018) under the terms of the Creative Commons Attribution 4.0 International Public License (CC-BY 4.0; https://creativecommons.org/licenses/by/4.0/) associated with that article. Illustrations were created in *Biorender*.

HF can be defined as the progressive inability of the heart to adequately pump blood to meet the demands of the body. We focus here on changes observed in CaV1.2 and RyR2 calcium channels during HF with reduced ejection fraction (HFrEF) which commonly occurs after cardiac injury eg. ischemia, or with sustained stress eg. hypertension. There remains a limited understanding of the effects of HF with preserved ejection fraction (HFpEF) on CaV1.2 and RyR2 channel function and regulation, hampered by difficulties in generating an accurate animal model (Eisner et al., 2020; Benitah et al., 2021). The current state of our knowledge of Cav1.2 and RyR2 channel reorganization in HF is summarized in Figure 3A and is discussed in detail in this section. Several investigators have demonstrated reduced efficiency of signal transduction between CaV1.2 channels and RyR2 during HF (Gomez et al., 1997; Louch et al., 2004). Reduced functional coupling between these two calcium channels has deleterious implications for cardiac EC-coupling. There are several possibilities to explain reduced functional coupling between these two channels for example: (1) remodeling of the t-tubule network and reduced number/length of dyadic junctions; (2) remodeling of the SR – with consequences for dyad integrity; (3) decreased expression and/or function of CaV1.2 and/or RyR2; (4) mis-localization or nanoscale reorganization of CaV1.2 and/or RyR2. We discuss each of these possibilities in more detail below.

### Remodeling of the T-Tubule Network

The effects of HF and myocardial infarction (MI) on the t-tubule network, dyad integrity, and SR positioning has been previously (Guo et al., 2013) and very recently reviewed (Setterberg et al., 2021). We refer the reader there for a detailed discussion of the architectural changes in myocytes during cardiac pathologies. However, since the t-tubules are critical ultrastructural components that constitute the setting for efficient CaV1.2 participation in EC-coupling, we briefly discuss these changes. Remodeling of the t-tubule network is observed as compensated cardiac hypertrophy transitions into HF, representing somewhat of a watershed event that tips the balance toward failure (Wei et al., 2010). The network undergoes extensive architectural remodeling during HF involving t-tubule loss (He et al., 2001; Balijepalli et al., 2003; Wei et al., 2010; Wu et al., 2012), disorganization (Louch et al., 2006; Song et al., 2006), dilation (Maron et al., 1975; Schaper et al., 1991; Kostin et al., 1998) and appearance of a larger number of longitudinally orientated tubules (Kaprielian et al., 2000; Song et al., 2006) reminiscent of architecture of immature myocytes (Lipsett et al., 2019). While we continue to focus on HFrEF here, it bears mentioning that myocytes from HFpEF patients and rats apparently do not display the typical HFrEF associated reduced t-tubule density, rather t-tubule density in HFpEF is either maintained or even increased relative to healthy myocytes (Frisk et al., 2021). Returning to the ‘immature’ phenotype idea, this refers to the fact that t-tubules are not present at birth in many species and only begin to develop around 10 days afterwards, initially in a disorganized fashion, only forming a mature network by around 3 weeks post-birth in mice (Hamaguchi et al., 2013), rats (Ziman et al., 2010), and rabbits (Haddock et al., 1999). In addition, the immature phenotype has been linked to the re-emergence of fetal gene expression profiles (Rajabi et al., 2007). For example, the formation of t-tubules and dyads coincides with the expression of JPH2 during development (Ziman et al., 2010), and knockdown of JPH2 during development inhibits t-tubule maturation (Reynolds et al., 2013), but in HF some groups have reported JPH2 expression is downregulated in a regression towards an immature phenotype (Minamisawa et al., 2004; Landstrom et al., 2007; Wei et al., 2010; Zhang et al., 2013; Guo et al., 2015). Similarly, the membrane curvature-mediating, and CaV1.2-targeting protein BIN1 has been demonstrated to play an early role in t-tubule biogenesis in muscle (Lee et al., 2002), but is downregulated in HF (Hong et al., 2012; Caldwell et al., 2014). We consider these two proteins and their role in t-tubule formation and stability, and dyad integrity in more detail below.

#### Role of Junctophilin 2

Junctophilin 2 is a junctional complex-forming protein that spans the width of the dyadic cleft with its hydrophobic C-terminal transmembrane domain anchored in the SR-membrane while its eight N-terminal Membrane Occupation Recognition Nexus (MORN) motifs bind phospholipids to form the sarcolemmal anchor (Takeshima et al., 2000). Acute knockdown of JPH2 in adult mice precipitates HF (Wei et al., 2010; van Oort et al., 2011), while AAV9-mediated restoration of JPH2 in TAC mice has been demonstrated to improve cardiac function, prevent t-tubule loss, and reduce aberrant Ca^2+^ leak (Reynolds et al., 2016). Loss of JPH2 effectively removes the tether that holds the SR and PM together and precipitates t-tubule drift away from the jSR leading to increased dyadic cleft width, and/or orphaning of RyR2. As a result of this aberrant physical distancing of RyR2 from CaV1.2 and the Ca^2+^ signal for CICR, dyssynchronous SR Ca^2+^ release occurs as RyR2 no longer open in unison during the AP but must wait a little longer for the Ca^2+^ trigger to reach them (Litwin et al., 2000; Louch et al., 2006; Song et al., 2006; Louch et al., 2013; Shiferaw et al., 2020). This dyssynchronous Ca^2+^ release does not favor efficient and uniform contraction and contributes to the contractile dysfunction of HF. Also contributing to the Ca^2+^ signaling irregularities, physical association of JPH2 with RyR2 stabilizes the closed state of the channel (van Oort et al., 2011). Loss of JPH2 thus eliminates this allosteric regulation, resulting in enhanced RyR2 activity and diastolic Ca^2+^ leak that impairs contractility and can trigger arrhythmias (Wang et al., 2014). Transgenic overexpression of JPH2 has been linked with enhanced RyR2 cluster size but reduced spark frequency, again attributed to the inhibitory influence of JPH2 on RyR2 opening (Munro et al., 2016).

Proteomic analyses have further revealed the serine/threonine protein kinase SPEG (striated muscle preferentially expressed protein kinase) as a binding partner of both JPH2 and RyR2, and thus another component of this dyadic protein complex (Quick et al., 2017). In that study, tamoxifen-inducible cardiac specific SPEG knockout in adult mice resulted in t-tubule and dyad loss that preceded HF and correlated with reduced SPEG-mediated phosphorylation of JPH2. This link between JPH2 phosphorylation and architectural remodeling remains correlative and the exact residue on JPH2 that is phosphorylated by SPEG remains to be determined. Another post-translational modification, namely S-palmitoylation has recently been revealed as a critical determinant of the tethering function of JPH2 to both the PM and SR/ER membranes (Jiang et al., 2019). A previous study reported that the palmitoylation status of BK potassium channels determines the ability of PKA to phosphorylate them, suggesting cross-talk between these two post-translational modifications confers conditional regulation of the channel (Tian et al., 2008). Future studies should examine the pathogenicity of JPH2 hypo-phosphorylation, and whether there is any cross-talk between JPH2 phosphorylation and the S-palmitoylation conferred stability of JPH2 tethers.

On the other hand, SPEG-mediated phosphorylation of RyR2 has been pin-pointed to residue S2367 and this post-translation modification inhibits diastolic Ca^2+^ leak, exerting a cardioprotective effect (Campbell et al., 2020). This effect was illustrated in a series of experiments from Xander Wehrens’ lab in which knock-in mice with constitutively activated RyR2-S2367D were more resistant to pacing-induced atrial fibrillation (AFib), while knock-in mice with a non-phosphorylatable alanine substitution at that same site (RyR2-S2367A) were more susceptible to AFib (Campbell et al., 2020). SPEG is also known to phosphorylate another critical regulator of cardiomyocyte Ca^2+^ homeostasis, SERCA2a at residue T484 to enhance SR Ca^2+^ reuptake (Quan et al., 2019). Furthermore, human loss-of-function mutations in SPEG are associated with dilated cardiomyopathy [as reviewed in (Campbell et al., 2021)] suggesting reduced SPEG activity is a risk factor for cardiac pathology.

Recent reports have suggested a role for JPH2-dependent recruitment of CaV1.2 channels to the dyads on the t-tubule plasma membrane *via* a physical interaction between the ‘joining region’ of JPH2 and an unknown portion of the CaV1.2 α_1C_ subunit (Gross et al., 2021; Poulet et al., 2021). In skeletal muscle, an association between CaV1.1 and the junctophilin isoforms JPH1 and JPH2 reportedly occurs through an interaction between the proximal C-terminal tail of the channel at amino acids 1,595–1,606 (Nakada et al., 2018), and amino acids 230–369 on JPH1 or 216–399 on JPH2 (Golini et al., 2011). In a chicken or the egg type of scenario it is difficult to determine whether mutant JPH2 fails to recruit CaV1.2 to the dyad because JPH2-CaV1.2 interactions are critical for dyad assembly or because the dyads are not there in the first place because the mutant JPH2 does not support them. Overexpression of JPH2 has been found to preserve t-tubule structure, an effect that was said to depend on the presence of cholesterol since membrane cholesterol depletion with methyl β-cyclodextrin (MβCD) counteracted the positive effects of JPH2 (Poulet et al., 2021). This idea remains to be tested in freshly isolated cells and is somewhat complicated by the fact that acute (Zhu et al., 2016) or chronic (Bryant et al., 2018b) disruption of lipid rafts and caveolae, and the mere act of myocyte culturing itself (Lipp et al., 1996; Louch et al., 2004; Pavlovic et al., 2010), each lead to t-tubule and dyad disruption. Furthermore, MβCD will deplete cholesterol from all cellular membranes (Zidovetzki and Levitan, 2007), not just the plasma membrane and will thus change patterns of gene transcription [e.g., *via* sterol response element binding protein, SREBP (Eid et al., 2017)] and mTOR activation (Castellano et al., 2017) with implications for metabolism, cellular growth, and contractility (Xu and Brink, 2016). Furthermore, previous studies have shown that alterations in cholesterol homeostasis lead to profound changes in the phospholipid landscape (Vivas et al., 2019; Kutchukian et al., 2021), and as mentioned above, JPH2 is known to associate with the plasma membrane *via* interactions between MORN motifs in its N-terminal with phosphatidylserine and phosphoinositol lipids PIP, PI(3,4,5)P_3_, and to a lesser extent PI(4,5)P_2_ (Takeshima et al., 2000; Bennett et al., 2013). Further investigations are needed to disentangle the fundamental role of JPH2 in dyad formation and integrity from a potential role in recruiting or retaining CaV1.2 at those sites.

As noted above, there have been some reports of JPH2 downregulation in failing myocytes from a thoracic-aortic banding induced pressure-overload rat model (Wei et al., 2010), transgenic mouse models of hypertrophic or dilated cardiomyopathies (Minamisawa et al., 2004), and human hypertrophic cardiomyopathy (Landstrom et al., 2007), dilated cardiomyopathy and ischemic cardiomyopathy (Zhang et al., 2013; Guo et al., 2015). However, sheep and ferret HF models display t-tubule loss with no change in JPH2 (Caldwell et al., 2014) and more recent investigations have reported no change in JPH2 expression in myocytes from human patients with idiopathic dilated cardiomyopathy (Hou et al., 2021) or in *jph2* transcript expression in left ventricular tissue samples from patients with dilated cardiomyopathy (Frisk et al., 2021). These conflicting results suggest that reduced JPH2 expression is not a universal feature of HF and may point to species and model-dependent differences, extending even to the different etiologies of human HF. These discrepancies in JPH2 expression also raise some questions about the proposed role of JPH2 as a key driver of t-tubule loss in HF.

#### Role of Bridging Integrator 1

We have already discussed the role of BIN1 in t-tubule formation (Lee et al., 2002; Frost et al., 2009; Hong et al., 2010), and the finding that BIN1 is downregulated in HF (Hong et al., 2012; Caldwell et al., 2014; see Figure 3A). Here we focus on evidence supporting a role of reduced BIN1 in the pathogenesis of HF. The link between reduced BIN1 expression and HF was first appreciated when *Bin1* gene deletion was found to cause perinatal lethality in newborn mice who succumbed to severe ventricular cardiomyopathy within the first 24–48 h of birth (Muller et al., 2003) while cardiac specific cre-recombinase driven *Bin1* knockouts were found to develop age-associated dilated cardiomyopathy (Laury-Kleintop et al., 2015).

Efficient EC-coupling in adult ventricular myocytes requires concentration of CaV1.2 channels at the t-tubules and this is achieved through channel delivery along BIN1-anchored microtubules (Hong et al., 2010, 2012). In HF, CaV1.2 expression at the t-tubules decreases as discussed in Section “Nanoscale Reorganization of CaV1.2” below, could this be linked to reduced transcriptional expression of BIN1? Super-resolution STORM imaging of CaV1.2 in ventricular myocytes revealed smaller clusters in *Bin1^+/−^* HET knockout myocytes compared to controls (Hong et al., 2014), while surface biotinylation biochemistry revealed reduced membrane expression of CaV1.2 in mouse cardiomyocytes after shRNA-mediated BIN1 knockdown (Hong et al., 2012). Furthermore, lentiviral-mediated BIN1 overexpression in hESC-CMs has been demonstrated to favor tubular membrane formation, and CaV1.2 clustering and recruitment to those BIN1 positive tubules to form functional CaV1.2-RyR2 containing CRUs (De La Mata et al., 2019). A separate study revealed AAV9-delivered BIN1 increases survival of mice with pressure-overload induced cardiomyopathy using the transverse aortic constriction (TAC) model, normalizing t-tubule membrane intensity and CaV1.2 and RyR2 distribution (Li et al., 2020). Collectively, these studies suggest BIN1 plays a role in enrichment and clustering of CaV1.2 on t-tubules and may stabilize CRUs with the implication that downregulation of BIN1 in HF is detrimental to t-tubular CaV1.2 recruitment, dyad integrity, and EC-coupling.

Interestingly, AAV9-delivered SERCA2a to failing rat hearts has also been reported to normalize t-tubule density, reduce Ca^2+^ dyssynchrony, and normalize BIN1 but not JPH2 levels suggesting that JPH2 was not required to restore a functional t-tubule network in that post-infarction rat model of left ventricular HF (Lyon et al., 2012) and implying a link between normalization of calcium signaling and reverse remodeling. It is tempting to speculate that elevations in diastolic Ca^2+^ levels during HF may reduce the ability of microtubules to polymerize and thus destabilize the t-tubule network and cause mis-localization of key ion channels and receptors. Indeed, elevated Ca^2+^ levels have long been known to trigger microtubule depolymerization (Weisenberg, 1972; Sandoval and Weber, 1978).

### Remodeling of the SR

The jSR structure and dynamics remains less studied than that of the t-tubule network although as the other half of the junctional membrane complex, its positioning is arguably of equal importance when considering the efficiency of cardiac EC-coupling. A recent study developed a new methodology to examine the mobility of the jSR using an AAV9-packaged photoactivatable GFP-tagged triadin (TRD-paGFP) that was transduced into cardiomyocytes *via* retro-orbital injections of live mice (Drum et al., 2020). Triadin localizes to the jSR where it anchors the Ca^2+^-binding protein calsequestrin (Casq2) to RyR2 (Zhang et al., 1997). Photoactivation of TRD-paGFP in discrete regions of interest permitted tracking of the jSR over time. While a subset of TRD-paGFP containing jSR elements were observed to remain statically secured in the vicinity of the t-tubule membrane, the majority were mobile and were frequently observed emerging toward, or retracting away from nearby t-tubules (Drum et al., 2020). Furthermore, jSR mobility and structural changes were regulated by microtubules and associated motor proteins in neonatal mouse, (Vega et al., 2011), adult mouse (Drum et al., 2020), and adult rat cardiomyocytes (Vega et al., 2011). The mobility was demonstrated to be reduced by co-transduction of a dominant negative motor-less form of kinesin 5b (Kif5b-DN) or by treatment with small hairpin RNA against dynein (Dnchc1-shRNA). Kinesin is a plus-end directed motor protein that carries cargo along microtubules away from the trans-Golgi while dynein is a minus-end directed motor protein that carries cargo in the opposite direction. In other cell types where fluorescent labeling of the ER is more easily achieved and distinguished from non-invaginating PMs, ER mobility has long been appreciated and indeed the ER is thought of as a highly dynamic organelle with ER-tubules tracking along microtubules as they undergo polymerization and growth or catastrophe and shrinkage (Pendin et al., 2011). Envisioning the jSR being propelled to-and-fro along microtubules brings-to-mind the Prosser lab’s elegant Airyscan acquired movies of microtubules buckling into spring-like structures as the cell contracts during systole and recoiling into the more familiar linear structures as the cell relaxes during diastole (Robison et al., 2016). If indeed the jSR tubules are anchored onto microtubules, what does this mechanical action do to the dyadic cleft? An elegant study from the Kohl lab recently demonstrated that t-tubule membranes experience the mechanical stresses and strains of systole and diastole with their membranes undergoing deformation on a beat-to-beat basis (Rog-Zielinska et al., 2021). It seems reasonable to assume the jSR suffers similar stresses and strains that could push it toward the t-tubule PM during contraction and pull it away again as the cell relaxes. The spacer function of JPH2 and the size of the cytosolic portion of RyR2 may act to prevent the total collapse of the dyadic cleft but the consequences of these dyadic deformations for EC-coupling remain to be fully explored.

There is little information on alterations in jSR motility during HF although disrupted SR networks have been observed in HF models (Pinali et al., 2013), and altered distributions of SR proteins are observed in hypertrophic myocytes (Hadipour-Lakmehsari et al., 2019). Microtubule growth and stability is decreased by the oxidative stress of MI, resulting in enhanced rates of microtubule catastrophe and impaired delivery of K_V_4.2 and K_V_4.3 potassium channels in mouse ventricular myocytes (Drum et al., 2016). Since jSR mobility relies on motor protein mediated transport along the microtubules, the effects of microtubule instability on jSR positioning and dynamics is an interesting prospect for future studies.

### Nanoscale Reorganization of CaV1.2

At the other side of the dyad, CaV1.2 channel localization and function is also altered in HF models. In the late 90s, cell-attached patch recordings from failing human left ventricular myocytes revealed enhanced CaV1.2 channel activity in what were likely overwhelmingly crest-located channel clusters but whole cell current density (*I*_Ca_) was unchanged compared to healthy control myocytes and it was prophetically posited that the ‘increased activity of superficial channels compensates for a reduced channel expression in the t-tubules’ (Schroder et al., 1998). A subsequent report from Clive Orchard’s lab interrogated the effect of detubulation on *I*_Ca_ in failing rat ventricular myocytes post coronary artery ligation (Bryant et al., 2015). Despite an overall preservation of *I*_Ca_ density in intact (tubulated) failing myocytes compared to healthy sham controls, detubulation revealed that the proportion of channels contributing to that current had shifted in their distribution from a predominantly t-tubular locale in health (24:76% crest:t-tubule ratio) to a more even distribution in failure (45.55% crest:t-tubule ratio; Bryant et al., 2015). Failing myocytes from TAC mice show a similar reduction in t-tubular *I*_Ca_ density but do not show an increase in density at the cell surface resulting in a diminished *I*_Ca_ compared to controls (Bryant et al., 2018a). This redistribution with fewer channels in the t-tubules, and sometimes more in the crest (depending on the species/HF model), combined with the alterations in t-tubule morphology in HF was linked to reduced expression of caveolin-3 (Bryant et al., 2018a) and has consequences for EC-coupling since the nanodomain Ca^2+^ signaling and functional coupling between t-tubular CaV1.2 and jSR RyR2 is impaired. Illustrating this, Ca^2+^ transients are reduced in amplitude in failing versus healthy sham controls despite similar *I*_Ca_ density reflecting a reduced ability of CaV1.2-mediated Ca^2+^ influx to trigger SR Ca^2+^ release, i.e., reduced EC-coupling gain (Gomez et al., 1997; Bryant et al., 2015). Early studies on changes in *I*_Ca_ with HF are reviewed here (Benitah et al., 2002) but we focus our remaining attentions on recent advances.

One relatively recent technical advance that has brought illuminatingly enhanced resolution to this field and enabled mapping and functional comparison of t-tubule versus crest-localized CaV1.2 channels are the super-resolution patch clamp experiments from the Gorelik lab. Using this approach, Bhargava confirmed other findings that CaV1.2 channels are concentrated (Kawai et al., 1999) and clustered on the t-tubules (Scriven et al., 2010; Dixon et al., 2015, 2017; Ito et al., 2019; Sato et al., 2019; Del Villar et al., 2021), finding that they were 4.3-times more likely to observe CaV1.2 activity from cell-attached patches in the t-tubules versus those in the crest (Bhargava et al., 2013). In subsequent studies on failing left ventricular myocytes CaV1.2 channels reportedly relocate from their usual preferred position on the t-tubules, to occupy new positions on the crest (surface sarcolemma; Sanchez-Alonso et al., 2016; see Figure 3A). In failing rat and human ventricular myocytes, the probability of landing the pipette on a patch of crest membrane with CaV1.2 channel activity was improved ~4-fold over that seen healthy cell crests (Sanchez-Alonso et al., 2016). Furthermore, the relocated crest-localized channels exhibited a 3 to 4.5-fold enhancement in *P*_o_ (Sanchez-Alonso et al., 2016) which was attributed to increased levels of constitutive channel phosphorylation due to elevated expression and activity of CaMKII in HF (Hoch et al., 1999; Kirchhefer et al., 1999; Zhang et al., 2003; Ai et al., 2005). Indeed, inhibition of CaMKII with KN-93 was seen to normalize the crest channel *P*_o_ to healthy levels and reduce arrhythmogenic Ca^2+^ oscillations (Sanchez-Alonso et al., 2016).

In failing right ventricular myocytes after left coronary artery ligation (an ischemic cardiomyopathy (ICM) model) the phosphorylating kinase was identified as PKA and the channel population displaying increased activity was on the t-tubules, not the crest (Medvedev et al., 2021). Interestingly, the phosphorylated channels on the t-tubules displayed overtly cooperative behavior that was inhibited by H-89, reminiscent of our previous work where we demonstrated PKA-dependent enhancement in channel cooperativity and stimulated insertion of multi-channel clusters into the t-tubule membrane [see Section (Ito et al., 2019; Del Villar et al., 2021)]. Whether PKA-triggered augmentation of t-tubular CaV1.2 channel expression similarly occurs in failing right ventricular myocytes remains to be tested. Overall, both crest and t-tubular CaV1.2 density was reduced in failing right ventricular myocytes, indicated by a reduced likelihood of recording CaV1.2 channel activity in cell-attached patches positioned in those locations in the failing myocytes compared to controls. Whether the likelihood of recording channel activity in t-tubule localized patches was further reduced by PKA-inhibition was not reported. Channel hyperphosphorylation and redistribution of channels has thus been reported in several cardiomyopathies including dilated cardiomyopathy (DCM), and ICM (Sanchez-Alonso et al., 2020). However, while CaMKII-mediated phosphorylation underlies the enhanced *P*_o_ of crest localized channels in DCM, PKA-mediated phosphorylation underlies the elevated *P*_o_ of t-tubule localized in ICM (Sanchez-Alonso et al., 2020) suggesting distinct nanodomain signaling pathways are differentially activated in these two pathologies.

### Nanoscale Re-Organization of RyR2

Work from Jon Lederer’s lab around the turn of the millennium revealed reduced functional coupling between CaV1.2 channels and RyR2 during HF secondary to hypertension (Gomez et al., 1997) or MI (Gomez et al., 2001). *I*_Ca_ was found to be maintained in HF myocytes but calcium transient amplitude and the ability to trigger Ca^2+^ sparks was reduced. Spatial remodeling was postulated as the cause of this reduced EC-coupling gain with three scenarios proposed, (1) mismatch where the t-tubule network remained unaltered by the CaV1.2 and RyR2 channels were misaligned; (2) increased width of the dyadic cleft; and (3) altered t-tubule networks resulting in ‘orphaned’ RyR2 whereby spatial reorganization of the t-tubule network would leave stranded jSR elements behind without juxtaposed CaV1.2 cluster partners. Remodeling of the t-tubule network had already been reported by Tim Kamp’s group (He et al., 2001) and so the ‘orphaned’ scenario gained favor and was later confirmed by Lederer’s group (Song et al., 2006). RyR2 orphaning was seen to occur in HF myocytes just as predicted, when the t-tubule network reorganized and became somewhat chaotic in appearance compared to the regular grid-like pattern observed in healthy myocytes. This leads to a loss of local control of RyR2 by CaV1.2. Cultured myocytes were seen to exhibit a similar t-tubule network reorganization and descendance into chaos as the culture period extended (Louch et al., 2004) and examination of the Ca^2+^ transient wavefront on line-scans from Ca^2+^ indicator loaded, paced myocytes revealed that cultured and failing myocytes displayed more frequent delays in activation of some RyR2 in agreement with an earlier report of dyssynchronous sparks in failing rabbit hearts post-MI (Litwin et al., 2000).

Orphaned, and/or smaller clusters of RyR are thought to contribute to the enhanced leak, and are posited to operate in an a silent or “invisible” mode where 1–5 RyR2 open in response to a lower intracellular Ca^2+^ trigger and produce no detectable spark (Sobie et al., 2006; Cheng and Lederer, 2008). While some SR Ca^2+^ leak occurs in healthy hearts, enhanced SR Ca^2+^ leak is a feature of many cardiopathies including MI, HF, and AFib (Shannon et al., 2003; Terentyev et al., 2008; Neef et al., 2010; Fauconnier et al., 2011; Fischer et al., 2014; Li et al., 2014). The increased leak reduces SR Ca^2+^ content leading to reduced amplitude transients, weaker contractions, and impaired relaxation due to elevated diastolic Ca^2+^ levels. The latter can lead to enhanced NCX activity precipitating arrhythmia (Molkentin et al., 1998; Chelu et al., 2009). Hyperphosphorylation of RyR2 by CaMKII has been proposed to underlie the enhanced activity at rest that generates the leak but this is not the full story as while CaMKII inhibitors can restore SR Ca^2+^ content, they do not fully normalize the amplitude of the Ca^2+^ transient (Ai et al., 2005) possibly because CaMKII also increases the fractional release of Ca^2+^ from the SR during systole (Li et al., 1997). These hyperactive CaMKII phosphorylated RyR2 are predominantly located in non-dyadic, ‘orphaned’ locations (Dries et al., 2018).

More recently, alterations in the nanoscale organization of RyR2 has been linked to the deviant Ca^2+^ signaling and reduced contractility in HF and AFib (MacQuaide et al., 2015; Kolstad et al., 2018). In a mathematical modeling study, RyR2 cluster size heterogeneity was linked to arrhythmogenic Ca^2+^ release from the SR (Xie et al., 2019). In their model, large clusters were found to open more easily, requiring a lower threshold Ca^2+^ trigger and generating a larger fractional release from the SR, resulting in locally reduced SR content due to larger, more frequent Ca^2+^ sparks and non-spark leak (Zima et al., 2010). Thus, although large clusters are predicted to increase diastolic Ca^2+^ levels, the likelihood that they will generate propagating arrhythmogenic Ca^2+^ waves is low because the low SR content they leave in their wake limits propagation through the cell. Small-sized clusters on the other hand were found to be less excitable, with a higher threshold for activation and lower leak, generating local regions of high SR Ca^2+^ load but again, a low propensity for Ca^2+^ wave propagation due to their low excitability. However, small clusters interspersed among larger ones, releases the brake on propagation as the small clusters help maintain the average SR Ca^2+^ concentration, allowing waves originating at large cluster hot-spots to propagate through the cell.

In HF and AFib, fragmentation of RyR2 clusters has been reported, which perhaps increases the heterogeneity of cluster sizes (MacQuaide et al., 2015; Kolstad et al., 2018; Sheard et al., 2019; Figures 3A,B). In the MacQuaide et al. study of a long-term paced sheep model of persistent AFib, although individual cluster size in atrial myocytes was unchanged by AFib, when one considers the CRU, defined as functionally grouped clusters within 150 nm edge-to-edge of one another, heterogeneity was present. AFib CRUs were more fragmented and contained more RyRs, dispersed over a larger area than healthy cell controls (MacQuaide et al., 2015). However, in a recent study of RyR2 clustering in diseased atrial myocytes from human AFib patients, no dispersion or reorganization of RyR2 was observed suggesting that RyR2 reorganization is not a necessary component of the Ca^2+^ leak and arrhythmogenicity associated with human AFib (Munro et al., 2021).

In the Kolstad et al study, myocytes isolated from post-infarction HF rats had a larger number of small RyR2 clusters than cells isolated from control rats. CRUs were also found to be smaller on average and more fragmented (Kolstad et al., 2018; Figure 3B). Mathematical modeling suggested that the smaller clusters generated more ‘silent’ or non-spark leak, and the cluster dispersal led to low fidelity Ca^2+^ sparks, slow spread of Ca^2+^, and overall dyssynchrony. In this special issue a study by Hou et al. also reported a trend toward smaller, more dispersed t-tubule adjacent RyR2 clusters and ~ 50% reduction in RyR2 expression in human myocytes isolated from heart samples obtained from patients with idiopathic dilated cardiomyopathy, compared to non-failing controls (Hou et al., 2021). These smaller clusters separated by larger distances were postulated to contribute to smaller Ca^2+^ transients and reduced amplitude of contraction. The rat and sheep studies discussed above suggest that altered nanoscale organization of RyR2 in cardiomyopathies can contribute to the altered kinetics of Ca^2+^ release and the disease phenotype in these species. Further investigations are needed to determine whether the same can be said for multi-factorial human cardiomyopathies given that human AFib does not appear to exhibit this phenomenon (Munro et al., 2021).

## Aging-Related Changes in Ventricular CaV1.2 Channel Function and Distribution

Cardiac health and function progressively decline with aging and in the ventricles, is associated with altered L-type calcium channel function and Ca^2+^ signaling (Zhou et al., 1998; Lakatta and Sollott, 2002; Fares and Howlett, 2010; Feridooni et al., 2015), and hyperphosphorylation (Kandilci et al., 2011) and hyper-glycation of RyR2 associated with enhanced SR leak (Ruiz-Meana et al., 2019). There is some evidence that, CaV1.2 channels undergo changes in their function and distribution in the aging ventricular myocardium in a manner somewhat reminiscent of the changes seen in HF. In aging (24 month old) rat ventricular myocytes, single channel recordings of CaV1.2 activity and ensemble currents from crest/surface sarcolemma localized cell-attached patches revealed increased channel number, *P*_o_, and functional availability compared to cells isolated from young 3-month old rats (Josephson et al., 2002). However, like the majority of HF studies, despite an increased number and *P*_o_ of a subpopulation of channels, whole cell *I*_Ca_ is generally preserved (Walker et al., 1993; Xiao et al., 1994) or decreased with aging (Feridooni et al., 2017; Kong et al., 2018). These results suggest that in aging ventricles the t-tubule localized channel population may be reduced with a reciprocal expansion of the crest population. Indeed detubulation experiments on ventricular myocytes isolated from aged, 24-month old mice revealed t-tubular *I*_Ca_ density was ~50% lower compared to 3-month old mice (Kong et al., 2018). However, in that same study surface *I*_Ca_ density was reportedly unchanged in apparent conflict with the findings of Josephson *et al* (Josephson et al., 2002), albeit with a different animal model (mice versus rats). Di8-ANEPPS visualization of t-tubule density by the same group revealed an ~12% decrease in t-tubule density with aging, with no apparent change in their orientation. A study of human left ventricular myocardium from patients aged between 19 and 75 also reported t-tubule network alterations and reduced JPH2 expression and clustering with aging, raising the question of dyad integrity (Lyu et al., 2021). These remain the only studies on t-tubule network integrity and channel localization in aged models and thus further investigation is required to reconcile the prior report of enhanced surface single channel activity with these findings. It could be that there are species-dependent differences in these parameters between mice and rats or that consideration of the frailty index could begin to tease out differences in the groups. The frailty index takes into account that people and animals age at different rates, in other words people/animals may be chronologically the same age but some will become ‘frail’ (more predisposed to adverse health outcomes) faster than others (Heinze-Milne et al., 2019).

## Concluding Remarks and Future Directions

It is apparent that nanoscale organization of CaV1.2 and RyR2 channels in clusters on their respective membranes contributes to the efficiency of Ca^2+^ release on the cellular level, and myocardial contractility on the tissue level. These variably-sized clusters are formed by stochastic self-assembly processes (Baddeley et al., 2009; Sato et al., 2019) and are dynamic and constantly maintained by cycles of endocytosis, delivery, and recycling that ensures a stable population of dyadic channels. However, the populations can be expanded when required as illustrated by the enhanced clustering of each channel that occurs during acute stress to augment Ca^2+^ release and contractility, contributing to the inotropic response. While aberrant reorganization, consisting of channel redistribution and cluster fragmentation occurs in heart failure and at least for CaV1.2, reorganization also appears to occur in aging too. However, much remains to be explored in the aging myocardium to fully elucidate any reorganization of CaV1.2 and RyR2 channels, and morphological changes in the t-tubule network that may have implications for EC-coupling gain.

Future studies should examine RyR2 clustering, organization, and SR remodeling in the aging myocardium. The field would benefit from a super-resolution patching study to assay channel activity at the t-tubules versus the crest. In addition, a study of the phosphorylation status of the channel subpopulations may provide insight into the enhanced channel activity observed at the crest (Josephson et al., 2002). Certainly, aging is associated with an enhanced sympathetic drive at rest and impaired reuptake of norepinephrine that would favor enhanced PKA activity (Kaye and Esler, 2005), yet the aging heart exhibits progressive age-associated loss of βAR responsivity (Lakatta et al., 1975; Stratton et al., 1992; Lakatta, 1993; White et al., 1994; Xiao et al., 1994; Cerbai et al., 1995; Xiao et al., 1998) which diminishes cardiac reserve capacity, rendering the heart less able to cope with acute stress and lowering the threshold for the development of HF (Strait and Lakatta, 2012). Deletion of the PKA regulatory subunit RIIβ has been reported to prolong lifespan and be cardioprotective in male but not female mice (Enns et al., 2009) while CaMKII activity/expression has not been well studied in the context of aging.

The full mechanistic picture of how CaV1.2 channels are recruited to the t-tubule membrane and retained there in dyadic nanodomains is still evolving with putative roles for JPH2, BIN1, Cav3, and lipid rafts as discussed herein. One tangible link between each of these proteins and even lipid rafts is the reliance on, or association with membrane lipids. JPH2 MORN motifs bind phospholipids to form the sarcolemmal anchor (Takeshima et al., 2000), BIN1 presence at the PM, and membrane curving-abilities are reliant on electrostatic interactions with phospholipids (Wu and Baumgart, 2014). PI(4,5)P_2_ reportedly accumulates in caveolae where the basic residues on caveolins are posited to play a role in sequestering it (Fujita et al., 2009) and lipid rafts have been proposed to serve as scaffolds of G-protein coupled receptors and PIP_2_ signaling (Myeong et al., 2021). Thus, alterations in the lipid profile of cardiomyocytes could impact everything from t-tubule formation, to CaV1.2 channel delivery, to dyadic integrity, and compartmentalization of receptor signaling. Future studies should examine the lipid and phospholipid profile of cardiomyocytes in health, disease, and aging.

To conclude, nanoscale organization of CaV1.2 and RyR2 calcium channels in clusters as a means to control the amplitude of Ca^2+^ entry, SR Ca^2+^ release and myocardial function is an emerging concept. This concept is not just applicable to the heart but has been identified as a regulatory mechanism in several other physiological processes including vascular tone (Pritchard et al., 2018), skeletal muscle mitochondrial biogenesis (Place et al., 2015), and neuronal excitability (Moreno et al., 2016). One hopes that the continued development of nanobodies and and affimers (Carrington et al., 2019; Dong et al., 2019), coupled with newer light microscopy approaches like MINFLUX (Balzarotti et al., 2017; Gwosch et al., 2020) and MINSTED (Weber et al., 2021) which each boast molecular scale resolutions on the order of 1–3 nm in x, y, and impressively ~2 nm axially (Gwosch et al., 2020) will aid continued exploration and new discoveries into how ion channel cluster organization and reorganization can influence physiological processes in health and disease.

## Author Contributions

The author confirms being the sole contributor of this work and has approved it for publication.

## Funding

This work was supported by an R01 grant from the US National Institutes of Health to RD (R01AG063796).

## Conflict of Interest

The author declares that the research was conducted in the absence of any commercial or financial relationships that could be construed as a potential conflict of interest.

## Publisher’s Note

All claims expressed in this article are solely those of the authors and do not necessarily represent those of their affiliated organizations, or those of the publisher, the editors and the reviewers. Any product that may be evaluated in this article, or claim that may be made by its manufacturer, is not guaranteed or endorsed by the publisher.
